# Do-(Not-)Mechanical-Circulatory-Support Orders: Should We Ask All Cardiac Surgery Patients for Informed Consent for Post-Cardiotomy Extracorporeal Life Circulatory Support?

**DOI:** 10.3390/jcm10030383

**Published:** 2021-01-20

**Authors:** Jorik Simons, Martje Suverein, Walther van Mook, Kadir Caliskan, Osama Soliman, Marcel van de Poll, Thijs Delnoij, Jos Maessen, Barend Mees, Roberto Lorusso

**Affiliations:** 1 Department of Cardio-Thoracic Surgery, CARIM School for Cardiovascular Diseases, Heart and Vascular Centre, Maastricht University Medical Centre (MUMC+), 6229 HX Maastricht, The Netherlands; jorik.simons@mumc.nl (J.S.); j.g.maessen@mumc.nl (J.M.); 2 Department of Intensive Care Medicine, Maastricht University Medical Centre (MUMC+), 6229 HX Maastricht, The Netherlands; martje.suverein@mumc.nl (M.S.); w.van.mook@mumc.nl (W.v.M.); marcel.vande.poll@mumc.nl (M.v.d.P.); thijs.delnoij@mumc.nl (T.D.); 3 School of Health Professions Education, Maastricht University, 6229 ER Maastricht, The Netherlands; 4 Academy for Postgraduate Medical Training, Maastricht University Medical Centre (MUMC+), 6229 HX Maastricht, The Netherlands; 5 Department of Cardiology, Heart Failure, Heart Transplantation and Mechanical Circulatory Support, Erasmus Medical Centre, 3015 GD Rotterdam, The Netherlands; k.caliskan@erasmusmc.nl; 6 Department of Cardiology, School of Medicine, National University of Ireland, H91 TK33 Galway, Ireland; osama.soliman@nuigalway.ie; 7 Department of Surgery, NUTRIM School for Nutrition and Translational Research in Metabolism, Maastricht University Medical Centre (MUMC+), 6229 HX Maastricht, The Netherlands; 8 Department of Cardiology, CARIM School for Cardiovascular Diseases, Heart and Vascular Centre, Maastricht University Medical Centre (MUMC+), 6229 HX Maastricht, The Netherlands; 9 Department of Vascular Surgery, CARIM School for Cardiovascular Diseases, Heart and Vascular Centre, Maastricht University Medical Centre (MUMC+), 6229 HX Maastricht, The Netherlands; barend.mees@mumc.nl

**Keywords:** extracorporeal life support, ELS, post-cardiotomy extracorporeal life support, PC-ECLS, do-(not-)mechanical-circulatory-support, D(N)MCS, ethics

## Abstract

Post-cardiotomy extracorporeal life support (PC-ECLS) has seen a substantial increase in use over the past 10 years. PC-ECLS can be a life-saving procedure and is mostly applied in the presence of unexpected, severe cardio-respiratory complication. Despite PC-ECLS being critical in allowing for organ recovery, it is unfortunately closely connected with an unpredictable outcomes, high morbidity, and, even in the case of cardiac function improvement, potential sustained disabilities that have a life-changing impact for the patient and his or her family. Since the decision to start PC-ECLS is made in an acute setting, there is often only limited or no time for self-determined choices. Due to the major impact of the intervention, it would be highly desirable to obtain informed consent before starting PC-ECLS, since the autonomy of the patient and shared-decision making are two of the most important ethical values in modern medicine. Recent developments regarding awareness of the impacts of a prolonged intensive care stay make this a particularly relevant topic. Therefore, it would be desirable to develop a structural strategy that takes into account the likelihood of such an intervention and the wishes and preferences of the patient, and thus the related autonomy of the patient. This article proposes key points for such a strategy in the form of a PC-ECLS informed consent, a do-(not-)mechanical-circulatory-support order (D(N)MCS), and specific guidelines to determine the extent of the shared decision making. The concept presented in this article could be a starting point for improved and ethical PC-ECLS treatment and application.

## 1. Introduction

Even though modern cardiac surgery is shifting to less invasive techniques, it is still associated with the need for rather aggressive and invasive procedures, particularly in the presence of cardiogenic shock. In adult cardiac surgery, from 2% to 6% of patients develop a pericardiotomy cardiogenic shock [1,2]. The majority can be weaned from cardiopulmonary bypass using inotropic drugs, but around 4% require temporary mechanical circulatory support, including post-cardiotomy (PC) extracorporeal life support (ECLS) [3,4,5,6]. In the last decade, ECLS has been established as an indispensable treatment for severe cardiopulmonary failure. It is used to support patients in refractory cardiogenic shock (secondary to PC shock or to myocardial infarction, myocarditis, or sepsis) or during extracorporeal cardiopulmonary resuscitation (ECPR). Furthermore, it can be a bridge to heart or lung transplantation and implantable, ventricular assist devices. The Extracorporeal Life Support Organization (ELSO) Registry has shown a substantial increase in the use of PC-ECLS over the past 10 years, with the highest use of ECLS seen in the United States [7,8].

Although PC-ECLS is a potentially life-saving procedure, it is associated with a high rate of (severe) complications: major bleeding (26.8% to 56.6%), vascular complications (i.e., compartment syndrome (7.3% to 14.5%) and limb ischemia potentially leading to amputation (2.3% to 9.3%)), neurological complications (9.9% to 17.7%), infections (19.5% to 44.0%), and permanent sensitive-motor limb deficit [9]. Due to the high risk associated with PC-ECLS, as well as its resource-intensiveness, such a treatment is confined to use as a last resort and as a bridge to diagnosis, treatment, and recovery. In the case of PC-ECLS, the survival-to-hospital-discharge rate is below 40%, and quality of life after discharge varies among patients [5,10,11].

The decision to proceed to PC-ECLS during the patient management is usually made in an acute setting, when unexpected cardio-pulmonary failure occurs and there is no possibility to consult the patient. While this is a frequently occurring and well-accepted circumstance in the treatment of unexpected life-threatening situations, complications that warrant the decision to proceed to PC-ECLS following elective surgery may be more predictable though not necessarily anticipated in many elective surgical cases. In particular, patients with multiple valve repair, endocarditis, or poor preoperative cardiac function are at risk of becoming ECLS-dependent following elective cardiotomy. In these cases, there may be ample time during the preoperative consultation to take into account the wishes and preferences of the patient, to safeguard their autonomy. With PC-ECLS becoming increasingly relevant for cardiac surgery patients, there is a need for a structural strategy to take into account the autonomy of the patient. Recent worldwide developments regarding the awareness of the impacts of a prolonged intensive care stay makes this a particularly relevant topic.

This narrative review discusses the available publications on this topic, and includes a discussion about why PC-ECLS should be included in the informed consent procedure for adult patients undergoing cardiac procedures, particularly in the presence of a high likelihood for PC-ECLS. The authors aimed at providing a comprehensive evaluation of the impact of PC-ECLS on the patient and family, and patient-specific risk predictions for PC-ECLS. Moreover, the role of the healthcare system and ethical context is addressed and discussed. Finally, the authors provide a proposal for a pre-operative informed consent contemplating a do-(not-)mechanical-circulatory-support (D(N)MCS) order.

## 2. Do-(not-)Mechanical-Circulatory-Support Orders?

Over the last four decades, it has become more common practice to discuss do-not-resuscitate (DNR) orders well in advance of a possible cardiac arrest [12,13,14]. This attitude was predominantly based on the growing awareness that not all that medically can be done, should be done [12]. In this contemporary era with rapidly evolving new technologies, much can be done to keep the patient alive regardless of the eventual quality of life. With the primum non nocere principle in medicine, responsible use of (new) technologies is the primary obligation for all clinicians. From the patient’s perspective, their quality of life is the ultimate goal, not just their survival through the use of a proposed therapy [15]. Furthermore, patients frequently do not want life-preserving measures at any cost, either based on their own experiences with relatives, their expectations of what quality of life will be afterwards, or how they want their life to come to an end.

Through these discussions, it has become more accepted to examine wishes surrounding end-of-life and it has given the patient a way to voice their preferences and strengthen their autonomy during the last years of life, or in the occasion of a life-threatening event requiring aggressive therapies associated with unpredictable outcomes. Moreover, it clarifies their wishes to their family, who often have to make difficult decisions when the occasion arises. Instead of thinking about what their relative’s wishes might be, they can focus on saying their farewells.

DNR orders are now routinely noted and checked on admission to hospitals, outpatient clinics, or during a general practitioner (GP) visit in many countries [12]. Modern DNR orders move away from the dichotomous ‘Yes/No’ question towards more extensive shared decision making (e.g., no escalation of care [16]). Cardiac surgery candidates go through a series of preoperative consultations for preparation and screening. Usually, these moments are used to inform and ask the patient about the planned surgical procedure, including risks of complications and death. However, the information about the permission or refusal of PC-ECLS in case of need—thus creating a do-(not-)mechanical-circulatory-support order—is usually neglected or very marginally explained. Even though the incidence of PC-ECLS is relatively low, the impact and risks of a complex intensive care unit (ICU) stay, on the patient and their family, are such that they should have time to think about it and decide whether or not they consent to the application of mechanical support, if necessary.

## 3. Impact on Family and Patient

After the decision to start PC-ECLS treatment, the patient and the family move into uncertain territory. Although the decision to start PC-ECLS is made by the medical professionals in an acute/emergency setting, once on PC-ECLS, some decisions are made with the relatives. As the family knows the patient and his or her preferences best, they represent his or her wishes in accordance with their shared decision making. Yet, while this sounds honorable, it can nevertheless put a major strain on the family, who are often experiencing an unexpected adverse event with unpredictable outcome. It is known that a patient’s relatives experience personal guilt, increased stress-levels while receiving “the call”, ICU stress (e.g., due to the appearance of the patient, perceived imminent risk of death when arriving at the ICU), and prolonged uncertainty about the survival [17,18]. Contributing factors for relatives to deal with the unusual situation include the presence or absence of medical knowledge, family support, peer support-forming inpatient families, hospital services, and informal/formal meeting groups [17]. Even in the long-term, caregivers of critically ill patients reported high levels of depressive symptoms [19].

Since PC-ECLS is associated with a large number of complications, the outcome of the treatment during the ICU stay is, as mentioned, highly unpredictable, with high likelihood of minor or major complications. These complications are relevant for the short- and long-term outcome. In the long-term, major physical complications may still severely impact the patient and their family [20,21]. Although intensive rehabilitation could improve long-term outcome, some complications still have a major, irreversible long-term impact. For example, when the treatment results in amputation or a permanent neurologic deficit, it is often no longer possible to continue with the same pre-intervention lifestyle, and patients and their families need to restructure their lives, including changing or quitting work and losing independence, which will decrease the quality of life of the patient and puts a major strain on the family [22,23].

It is also well-known that critical illnesses and surgical procedures can have a major impact on the patients’ psychological state [24]. Since PC-ECLS treatment is only utilized in critically ill patients, such a procedure will influence the psychological well-being of the patient even more. Long-time follow-up shows that significant anxiety is present in one third to half of the patients after ECLS, depression in one fourth, and that only half of the patients return to work [25,26,27,28,29,30,31]. Furthermore, survivors have high rates of post-traumatic stress symptoms (PTSS) and post-traumatic stress disorder (PTSD) [25,29,30,32]. PTSD risk factors include age, mechanical ventilation, administration of drugs, adverse outcomes like delirium or agitation, and being “awake” during ECLS [32]. It is worth mentioning that the high prevalence of the outcome measures mentioned above might also be due to prolonged ICU length of stay [28,33]. Concerning the impact on the patient, McDonald et al. found a patient need for education, improved coordination of care and follow-up scheduling, and additional mental health resources [26].

The expected psychological and functional well-being of the patient is highly relevant since patients have different opinions on quality of life. Patients with high standards for quality of life will approach D(N)MCS orders differently in comparison to that of patients with lower standards. It is therefore important to get an overview of the psychological impact of PC-ECLS to give patients a perspective on what they can expect.

In the end, a PC-ECLS treatment has a major impact on the patients and their families and it would therefore be understandable if a patient might choose not to try to extend his or her life with PC-ECLS, even with a likelihood of recovery, and/or wants to discuss the potential adverse outcomes with their family members. Of course, it is difficult to predict the psychological and functional outcome of the treatment since it is determined by the clinical situation and a variety of patient characteristics. The majority of patients have never experienced a prior ICU stay, which makes it difficult to predict or estimate the impact of the psychological effect of the stay on the patient. Therefore, being fearful of the psychological outcome should, in most patients, not be a prime reason to initiate a D(N)MCS order. Although mortality is an important outcome measure, discharge from the intensive care unit with a marginal quality of life could still be considered an unsuccessful treatment. Table 1 gives an overview of functional and psychological patient outcomes after ECLS.

## 4. PC-ECLS and the Health Care System

Besides the patient’s preference for whether or not he/she wants to try to extend life with PC-ECLS towards organ recovery or as a bridge to other more advanced therapies, the actual possibilities (resources and expertise) for PC-ECLS in the treating hospital should also be taken into account. If the cardiothoracic center does not have experience with PC-ECLS, it might be advisable to consider other options if the likelihood of PC-ECLS is relatively high. Barbaro et al. showed lower odds of mortality at hospitals treating more than 30 adult ECMO cases annually [34]. Therefore, centers need to define their competence with PC-ECLS treatment and be transparent about their expertise with the patient. Limited experience in such a setting, therefore, might lead to a higher rate of transient or permanent injury of the treated patient, or other circumstance that might be either explained to the patient or be the reason for patient referral to more experienced centers.

If transport to another hospital is an option, this should be discussed with the patient, since it might not be in accordance with his or her treatment goals or expectations. Transportation will bring along additional risk and stress, particularly if the family would not be able to visit the patient after transportation; the patient needs to consider all this when deciding if they should go to another hospital with higher access to such therapies. If there is no feasible PC-ECLS treatment available in the treating hospital, or a possibility to transport the patient to a PC-ECLS center, it might be considered unethical to operate on patients with a high likelihood of needing PC-ECLS, and this should be openly disclosed to the patient and relatives.

## 5. Informed Consent and Patient Autonomy

Informed consent is, ethically and legally, used to practice medicine with respect to patient autonomy. It consists of (1) the physician informing the patient about the medical exam or procedure and (2) the patient giving permission for the discussed exam or procedure. Required elements for the informed consent discussion include (1) nature of the procedure, (2) risks associated with the procedure, (3) benefits associated with the procedure, (4) reasonable alternatives, (5) risks of the alternatives, and (6) benefits of the alternatives [35,36]. Ensuring that the patient understands these elements is also required. Without permission from the patient, the patient’s integrity is unauthorizedly breached.

Autonomy is one of the four basic principles in medical ethics—next to justice, beneficence and non-maleficence—and a central value in modern medicine [37]. It is defined as the right or possibility for the patient to make self-determined choices. Since the decision to start PC-ECLS is made in an acute setting, there is no time for self-determined choices. Thus, in most cases, PC-ECLS is based on an opt-out principle; the patient will be treated unless pre-discussed and documented refusal for PC-ECLS has been obtained. Therefore, it would be desirable to develop a structural shared decision making strategy that takes into account the wishes and preferences of the patient and maintains the autonomy of the patient. A D(N)MCS order could be that structural strategy.

## 6. What Should the Informed Consent Look Like?

Developing an informed consent for PC-ECLS and a D(N)MCS order is challenging. The most difficult part of the informed consent procedure is to ensure the patient and family have sufficient and understandable information, since it is essential to making a deliberate decision. Although it is highly desirable, patients—and even the clinicians—will never be able to completely understand the situation. In addition, when comparing PC-ECLS to resuscitation, resuscitation is a more familiar subject to the average person and many have considered or have been asked to consider their DNR status. PC-ECLS is, however, an unknown treatment that could pave the way for a rushed or not-supported decision. If patients are familiar with ECLS via newspapers or the internet, they might have an over-optimistic portrayal of ECLS, since it is known that these media show unrealistic survival rates [38]. So, it would be crucial to provide patients with sufficient and real-world information on which they can base their decision and give them enough time to consider, discuss, and ask follow-up questions. As with all shared decision making, clinicians should take into account the patient’s knowledge and adapt to their capabilities (e.g., level of education). If the patient is incompetent, the clinical team should turn to the (legal) representative.

The shared decision making should include the basic elements for informed consent: explanation of the nature of PC-ECLS, risks and benefits associated with PC-ECLS, and the alternatives and their risks and benefits. The physical and psychological complications need to be identified as risks and the chance of recovery and survival should be discussed. Moreover, the likelihood of PC-ECLS for the surgery and the possibility of transportation should be explained. It is important to emphasize that: (1) clinical situations at PC-ECLS implantation vary, (2) underlying life-threatening conditions may alter the response to PC-ECLS, and (3) that short- and long-term outcomes vary. Treatment goals regarding PC-ECLS situations, like a low likelihood of success (bridge to no recovery), are also relevant to discuss since PC-ECLS does not have an endless duration [39,40]. Furthermore, there is a trend to apply ECLS support prophylactically to enhance the perioperative course in critically ill patients in some circumstances: this situation deserves specific attention and discussion with the patient and the family beforehand. It is important to mention the difference between prophylactic and bail-out ECLS.

Since the likelihood of PC-ECLS varies among surgical procedures, it might not be recommended to combine informed consent for PC-ECLS on one hand, and a D(N)MCS order on the other, in the same patient prior to a surgical procedure. If the likelihood is relatively low, like with most routine cardiac surgery patients, it could redundantly increase the strain on the patient before surgery and might affect the patient’s willingness to undergo surgery. Clinicians should anticipate stress and fear by the patient and their family and guide the patient preoperatively. Therefore, limiting the extent of the shared decision making could balance the strain on the patient with the expected risk for PC-ECLS and its consequences. Moreover, if PC-ECLS is highly likely in a certain surgical procedure, a D(N)MCS order would be highly recommended, and not defining the wishes of the patient beforehand could even be considered unethical.

After informing the patient correctly and completely, the patient should give his or her view on PC-ECLS treatment and decide whether or not a D(N)MCS order should apply. Since it is not an easy decision for the majority of patients, the timing of the shared decision making is essential and could have a significant impact on the patient’s choice. To give patients the chance to think about PC-ECLS, and make a self-directed decision, sufficient time is needed between the information and the decision. So, it is best to address PC-ECLS treatment in an early phase to allow for deliberation and questions, and let the patient express his or her choice shortly before the surgery. If patients do not get time for deliberation and questions, there is a high chance for rushed decisions. It is also important to avoid wait-and-see approaches where the ultimate decision still lays in the hands of the family. This would enhance the strain on the family and miss the opportunity for informed consent. In the case of emergency surgery, the informed consent procedure would be difficult, if not impossible. In these cases, the current opt-out system is probably the most convenient since mentioning PC-ECLS would be difficult or impossible.

To conclude, proper and timely information and planning is highly important to make shared decision making and informed consent for PC-ECLS and D(N)MCS orders work. Since the likelihood of PC-ECLS varies among cardiac surgical procedures, not every patient needs the same amount of information or a D(N)MCS order. Table 2 gives an overview of the most important points and circumstances regarding preoperative informed consent for PC-ECLS.

## 7. Likelihood of PC-ECLS

**Figure 1:**
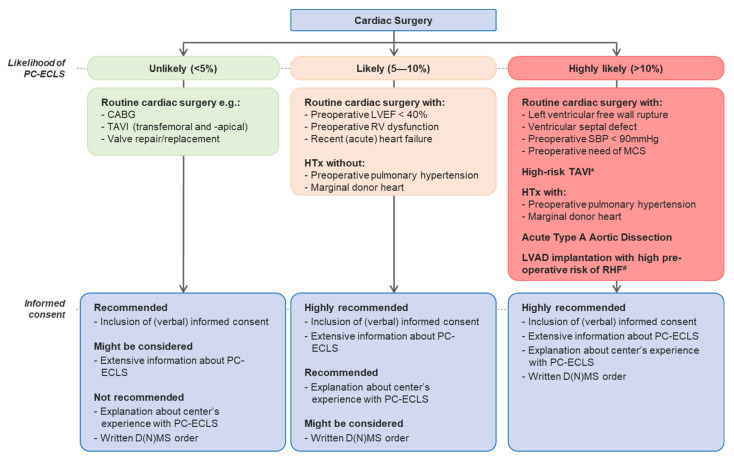
Recommendations regarding informed consent for PC-ECLS. Abbreviations: CABG: coronary artery bypass grafting; TAVI: transcatheter aortic valve implantation; PC-ECLS: post-cardiotomy extracorporeal life support; D(N)MCS: do-(not-)mechanical-circulatory-support; LVEF: left ventricular ejection fraction; RV: right ventricle; HTx: heart transplant; SBP: systolic blood pressure; MCS: mechanical circulatory support; LVAD: left ventricular assist device; RHF: right heart failure. Captions: *Dilated ascending aorta, bicuspid aortic valve, coronary anatomy, small and calcified aortic annulus, redo operation, see: Raffa GM, et al. In-hospital outcomes after an emergency or prophylactic veno-arterial extracorporeal membrane oxygenation during transcatheter aortic valve implantation: a comprehensive review of the literature. Perfusion. 2019; #RHF denotes: right heart failure, see: Soliman O et al. Derivation and Validation of a Novel Right-Sided Heart Failure Model After Implantation of Continuous Flow Left Ventricular Assist Devices: The EUROMACS (European Registry for Patients with Mechanical Circulatory Support) Right-Sided Heart Failure Risk Score. Circulation 2018.

As discussed earlier, the likelihood of PC-ECLS should be included in the shared decision making and will determine the extent and nature of the informed consent procedure (e.g., conveying information verbally, providing additional education via an information leaflet, asking for written informed consent) and whether or not a D(N)MCS is recommended. If PC-ECLS is contraindicated, for example due to age, and there is a high likelihood of PC-ECLS, it is also recommended to inform the patient about the contraindication. Contraindications are defined in the most recent guidelines. Figure 1 shows the proposed decision-making regarding the informed consent for PC-ECLS.

The likelihood of PC-ECLS is divided into three categories: unlikely (<5%), likely (5–10%), and highly likely (>10%). Table 3 gives an overview of the likelihood of necessity to initiate PC-ECLS for different cardiac procedures. In the case of “Preoperative left ventricular ejection fraction (LVEF) < 40%”, “Preoperative right ventricle (RV) dysfunction” and “Preoperative systolic blood pressure (SBP) < 90 mmHg”, the available research is limited, but it suggests poor PC-ECLS outcome or increased likelihood [41,42,43]. Retrospective research will be necessary to get a complete overview of the prevalence of PC-ECLS rates in cardiac surgery procedures. The addition of major outcomes like in-hospital mortality and the most relevant complications will give clinicians the data to correctly present patients with their risks and benefits.

The informed procedure was divided into four categories: (1) non-written inclusion to informed consent procedure; (2) extensive information about PC-ECLS; (3) explanation about center’s experience with PC-ECLS and (if applicable) risk of transport; and (4) inclusion of (written) D(N)MCS order. The first category consists of naming PC-ECLS shortly in the current preoperative informed consent and the second category contains the subjects under “What should the informed consent look like?” The third category also includes an explanation about the center’s experience with PC-ECLS and the latter category includes a D(N)MCS order. Recommendations ranged from: “not recommended”, “might be considered”, “recommended” to “highly recommended”. For each likelihood category, a set of recommendations applies.

## 8. Conclusions

PC-ECLS can be a life-saving procedure, but unfortunately it is closely and potentially connected with unpredictable outcomes, high morbidity, and, rarely, permanent patient complications inducing a life-changing moment for the patient and his or her family. Due to the major impact of the intervention, it would be highly desirable to practice shared decision making and obtain informed consent before starting the treatment. However, due to acute situations, this is often not possible, and an opt-out principle is usually used where the clinician makes the decisions. According to the type of procedure and risks of the surgical candidate, as well as the center characteristics in relation to PC-ECLS, a more comprehensive and structured approach for dedicated shared decision making and informed consent should be applied, providing a known and agreed path in case of need. A scoring system could potentially further refine this process. The proposed categorization of likelihood may represent the first step towards a solid version of informed consent for PC-ECLS and D(N)MCS orders.

## Figures and Tables

**Table 1:** Overview of psychological and functional outcomes after ECLS.

Outcome	Author (Reference)	ECLS-Mode	Main Indication	Total Patients, N	Median Time to ECLS, Years	Prevalence, %
Clinically significant anxiety (HADS)	Muller et al. [25]	VA	AMI	41	2.7	34
	McDonald et al. [26]	VV	ARF	42	1.2	48
	Schmidt et al. [29]	VV (95%)	ARDS	67	1.4	34
	O’brien et al. [30]	VV	ARF	13	3.0	54
	Orbo et al. [31]	VA (75%)	C (60%), P (25%) ECPR (15%)	20	6.5 (mean)	15
Anxiety/depression (EQ-5D)	Wang et al. [28]	VV	ARDS	24	1.0	None: 58 Moderate: 21 Extreme: 21
Depression (HADS )	Muller et al. [25]	VA	AMI	41	2.7	20
	McDonald et al. [26]	VV	ARF	42	1.2	26
	Schmidt et al. [29]	VV (95%)	ARDS	67	1.4	25
	O’brien et al. [30]	VV	ARF	13	3.0	15
	Orbo et al. [31]	VA (75%)	C (60%), P (25%) CPR (15%)	20	6.5 (mean)	0
At risk for PTSD (IES(-R))	Muller et al. [25]	VA	AMI	41	2.7	5
	Schmidt et al. [29]	VV (95%)	ARDS	67	1.4	16
	O’brien et al. [30]	VV	ARF	13	3.0	23
Pain/discomfort (EQ-5D)	Wang et al. [28]	VV	ARDS	24	1.0	None: 71 Moderate: 29 Extreme: 0
Katz ADL and Lawton IADL	McDonald et al. [26]	VV	ARF	42	1.2	No deficiencies: 62 Mild/moderate: 19 Severe: 19
Return to work	Orbo et al. [31]	VA (75%)	C (60%), P (25%) CPR (15%)	20	6.5 (mean)	50

Abbreviations: ADL: activities of daily living; AMI: acute myocardial infarction; ARDS: acute respiratory distress syndrome; ARF: acute respiratory failure; C: circulatory; CPR: cardiopulmonary resuscitation; ECLS: extracorporeal life support; ECMO: extracorporeal membrane oxygenation; HADS: hospital anxiety and depression scale; IADL: instrumental activities of daily living; IES(-R): impact of event scale (-revised); P: pulmonary; PTSD: post-traumatic stress disorder; VA: veno-arterial; VV: veno-venous; yr.: year.

**Table 2:** Key points for PC-ECLS informed consent and D(N)MCS orders.

Preoperative Informed Consent for PC-ECLS and D(N)CS Orders	
Increasing Knowledge of the Patient	
- General concept of PC-ECLS - Impact of PC-ECLS - Risks and benefits of PC-ECLS - Likelihood of PC-ECLS	- Option to decline PC-ECLS - Unpredictability of PC-ECLS - Expertise of hospital with PC-ECLS - Likelihood of success
Timing of D(N)MCS order	
- Phase 1 (information): as early as possible - Phase 2 (decision): relatively shortly—term before the surgery - Avoiding ‘wait-and-see’ approaches—minimize the strain on the family	

Abbreviations: D(N)MCS: do-(not-)mechanical-circulatory-support; PC-ECLS: post-cardiotomy extracorporeal life support.

**Table 3:** Likelihood of PC-ECLS with regards to several surgical procedures.

Procedure	Author (Reference)— Year of Publication	Total Patients, N	Prevalence of PC-ECLS Use, %	In-Hospital Mortality, %
Unlikely (<5%)				
CABG (isolated)	Biancari et al. [44]—2017	24,527	VA-ECMO: 0.6	64.2
	Raffa et al. [45]—2019	5115	Emergency VA-ECMO: 1.3 (TF and TA)	
TAVI	Trenkwalder et al. [46]—2017 Trenkwalder et al. [46]—2017	1424 370	Emergency VA-ECMO, TF: 1.5 Emergency VA-ECMO, TA: 3.0	39 Overall: 45.5
SAVR				
Non-differentiated	Mäkikallio et al. [47]—2019	4333	ECMO/IABP: 1.8	N/A
Without recent AHF	Jalava et al. [48]—2019	3757	ECMO/IABP: 1.3	N/A
Likely (5–10%)				
SAVR (with recent AHF)	Jalava et al. [48]—2019	484	ECMO/IABP: 5.6	N/A
HTx	Phan et al. [49]—2017	11,555	Overall MCS: 6.0	VA-ECMO: 44.4
			VA-ECMO: 4.8	
			RVAD: 0.7	RVAD: 62.1
			BiVAD: 0.5	BiVAD: 14.3
			LVAD: 0.1	LVAD: 37.5
Highly likely (>10%)				
Ventricular septal defect	Huang et al. [50]—2015	47	Preoperative IABP: 72.3 Preoperative VA-ECMO: 12.8	N/A N/A
HTx (marginal donor heart; LVEF < 45)	Listijono et al. [51]—2011	9	VA-ECMO: 89	12
Post-MI LVFWR	Formica et al. [52]—2017	35	Overall ECLS: 60 IABP: 28.6 VA-ECMO: 31.4	Overall: 43 IABP: 30 VA-ECMO: 50
aTAAD	Lin et al. [53]—2017 Wang et al. [54]–2019	162 246	VA-ECMO: 12.3 VA-ECMO: 2.8	65 14.3
LVAD implantation (isolated)	Riebandt et al. [55]—2017	154	VA-ECMO: 21	25

Abbreviations: AHF: acute heart failure; aTAAD: acute type A aortic dissection; BiVAD: biventricular assist device; CABG: coronary artery bypass grafting; ECMO: extracorporeal membrane oxygenation; HTx: heart transplant; IABP: intra-aortic balloon pump; LVAD: left ventricular assist device; LVEF: left ventricular ejection fraction; LVFWR: left ventricular free wall rupture; N/A: not available; RVAD: right ventricular assist device; Post-MI: post-myocardial infarction; SAVR: surgical aortic valve replacement; TA: transapical; TAVI: transcatheter aortic valve implantation; TF: transfemoral; VA: veno-arterial.

## Data Availability

Not applicable.
